# Efficacy and safety of first-line immune checkpoint inhibitor combination therapies in patients with advanced esophageal squamous cell carcinoma: a network meta-analysis

**DOI:** 10.3389/fonc.2024.1369848

**Published:** 2024-11-12

**Authors:** Chenglong Wang, Tongze Cai, Jiangcun Wei, Ying Huang, Lin Xiao, Tong Li, Zujie Qin

**Affiliations:** ^1^ Guangxi International Zhuang Medicine Hospital Affiliated to Guangxi University of Chinese Medicine, Nanning, China; ^2^ Graduate School, Guangxi University of Chinese Medicine, Nanning, China

**Keywords:** network meta-analysis, first-line immune checkpoint inhibitor combination therapies, advanced esophageal squamous cell carcinoma, efficacy, safety

## Abstract

**Background:**

We performed a network meta-analysis of phase III trials to compare the efficacy and safety of first-line regimens for patients with advanced esophageal squamous cell carcinoma (ESCC).

**Methods:**

A systematic review and Bayesian network meta-analysis were conducted by retrieving relevant literature from PubMed, Embase, the Cochrane Library, and the Web of Science. We included published sources of randomized clinical trials comparing immunotherapy combinations for treating advanced ESCC.

**Results:**

We analyzed seven studies involving eight immunotherapy combinations and 4688 patients. For patients without programmed death-ligand 1 (PD-L1) selection, it was found that the combination of toripalimab and chemotherapy provided better overall survival than chemotherapy alone (hazard ratio = 0.58, 95% confidence interval (CI) 0.43-0.78). Compared with chemotherapy alone, Sintilimab or camrelizumab plus chemotherapy seemed to achieve the best progression-free survival (hazard ratio = 0.56, 95% CI 0.46-0.68). Nivolumab plus chemotherapy appeared to provide the best objective response rate, with significant differences versus chemotherapy alone (odds ratio = 0.49, 95% CI 0.38-0.64). Nivolumab plus ipilimumab resulted in a relatively lower incidence of adverse events of grade ≥3 than other regimens.

**Conclusions:**

The combination of immune checkpoint inhibitors (ICIs) with chemotherapy provided a high probability of more effective treatment in comparison with chemotherapy alone for patients with advanced ESCC. Toripalimab and sintilimab plus chemotherapy were ranked as providing the highest OS and PFS benefit in the first-line setting, respectively.

## 1 Introduction

Esophageal cancer is the seventh most common cancer worldwide, with an estimated 604,100 new cases diagnosed in 2020 (1). Esophageal cancer causes more than half a million cancer-related deaths worldwide each year, with squamous-cell carcinoma accounting for approximately 85% of cases (2). More than two-thirds of patients with esophageal cancer are diagnosed with advanced or metastatic disease and the overall 5-year survival rate (OS) is only ~20%. For comparison, the most common gastrointestinal tumor and colorectal cancer patients have a 5-year OS of 65% (3). The standard treatment of patients with advanced or metastatic esophageal squamous cell carcinoma (ESCC) is platinum agents combined with a fluoropyrimidine or paclitaxel (4), which has not provided very satisfactory survival benefits over the past few decades, with median overall survival rarely surpassing 10 months (5).

Currently, with the further development of immunotherapy, more and more clinical studies on immune checkpoint inhibitors (ICIs) have been conducted, among which programmed death receptor 1 (PD-1), PD-ligand 1 (PD-L1), and cytotoxic T lymphocyte-associated protein (CTLA-4) inhibitors have achieved success in the treatment of ESCC. Compared with chemotherapy, immunotherapy combined with chemotherapy not only prolongs survival in second-line treatment (6), but also performs well in first-line treatment. Various immune checkpoint inhibitors (ICIs) have shown effective antitumor activity. For example, in the KEYNOTE-590 trial, compared with placebo plus chemotherapy, pembrolizumab plus chemotherapy improved overall survival in patients with previously untreated, advanced ESCC (7). Moreover, nivolumab, toripalimab, ipilimumab, camrelizumab, and sintilimab also significantly improved the OS and the progression-free survival (PFS). Therefore, immunotherapy combined with chemotherapy is expected to achieve long-term anticancer effects in the treatment of ESCC.

Although meta-analysis of several randomized clinical trials (RCTs) demonstrated remarkable increases in OS with immuno-chemotherapy compared with doublet chemotherapy in the first-line treatment of advanced or metastatic ESCC, there was no head-to-head comparison of different immuno-chemotherapy combination treatments in the first-line setting of ESCC. For this reason, we performed a systematic review and network meta-analysis to compare and rank the efficacy and safety of different ICIs plus chemotherapy for patients with ESCC, and to decide on the best treatment for patients grouped according to clinically relevant subgroups, such as gender, age, and PD-L1 expression.

## 2 Methods

This network meta-analysis was performed in accordance with the preferred reporting items for systematic reviews statement for network meta-analysis (PRISMA-NMA) (8).

### 2.1 Data search strategy

We searched PubMed, Embase, the Cochrane Library, and the Web of Science to identify relevant articles published up to 30 August 2023 using the search terms ‘pembrolizumab’ OR ‘nivolumab’ OR ‘tislelizumab’ OR ‘toripalimab’ OR ‘ipilimumab’ OR ‘serplulimab’ OR ‘camrelizumab’ OR ‘sintilimab’ OR ‘immunotherapy’ OR ‘PD-1’ OR ‘PD-L1’ OR ‘CTLA-4’ and ‘esophageal squamous cell carcinoma’ OR ‘esophageal neoplasms’ and ‘advanced’ OR ‘metastatic’ and ‘first-line’ and their medical subject head (MeSH) terms within the limitation of randomized controlled trials.

### 2.2 Inclusion criteria

The eligible studies were individually verified by two experienced investigators according to the following criteria: (1) the study design must be a phase III randomized clinical trial (RCT); (2) patients must be diagnosed with advanced or metastatic esophageal carcinoma and not previously treated; (3) the study must have investigated two or more groups treated with immunotherapy in comparison with or without combined chemotherapy; (4) one or more of the following outcomes must have been measured: overall survival (OS), progression-free survival (PFS), objective response rate (ORR), adverse events of grade ≥3 (Grade ≥3 AEs).

### 2.3 Exclusion criteria

Studies were excluded if they met at least one of the following exclusion criteria: (1) RCTs with ambiguous clinical outcomes; (2) trials comparing treatments that have not been approved by the FDA or equivalent; (3) single-arm studies, retrospective studies, reviews, or meta-analyses.

### 2.4 Data extraction and quality assessment

The extracted information included first author, year of publication, study design, sample size, patients’ ages and sex distribution, PD-L1 expression, and Eastern Cooperative Oncology Group performance status score. In addition, the clinical outcomes extracted included hazard ratios (HRs) with corresponding 95% confidence intervals (95% CIs) for OS and PFS and the incidence of ORR, and AEs of grade ≥3.

The risk of bias in each included study was assessed using the Cochrane Collaboration tool (9), which assigns grades of “high risk”, “unclear risk”, or “low risk”.

### 2.5 Statistical analysis

The primary outcomes were OS and PFS, and the secondary outcomes were ORR and AEs of grade ≥3. For network meta-analysis, Stata (17.0) was used to generate network plots to illustrate the directly comparative and indirectly comparative relationships among the treatment options included in the trials (10). Statistical analysis was performed using the ‘gemtc’ package of ‘R’ software (version 4.3.1). A fixed-effects network meta-analysis was conducted using a Bayesian framework. Using ‘JAGS’ for Markov-chain Monte Carlo sampling, we ran 50,000 simulations with the first 20,000 as the burn-in period and a thinning interval of 1. The models were checked for convergence using the Gelman-Rubin diagnostic. We evaluated the global heterogeneity between treatment effects across all studies by using the I^2^ statistic, with values under 25%, between 25% and 50%, and over 50%, respectively (11). The surface under the cumulative ranking curve (SUCRA) was applied to rank regimens based on their probabilities of being the optimal choice for each outcome (12). The higher the SUCRA value, the higher the probability of greater efficacy (13).

## 3 Results

### 3.1 Systematic review and characteristics

**Figure 1:**
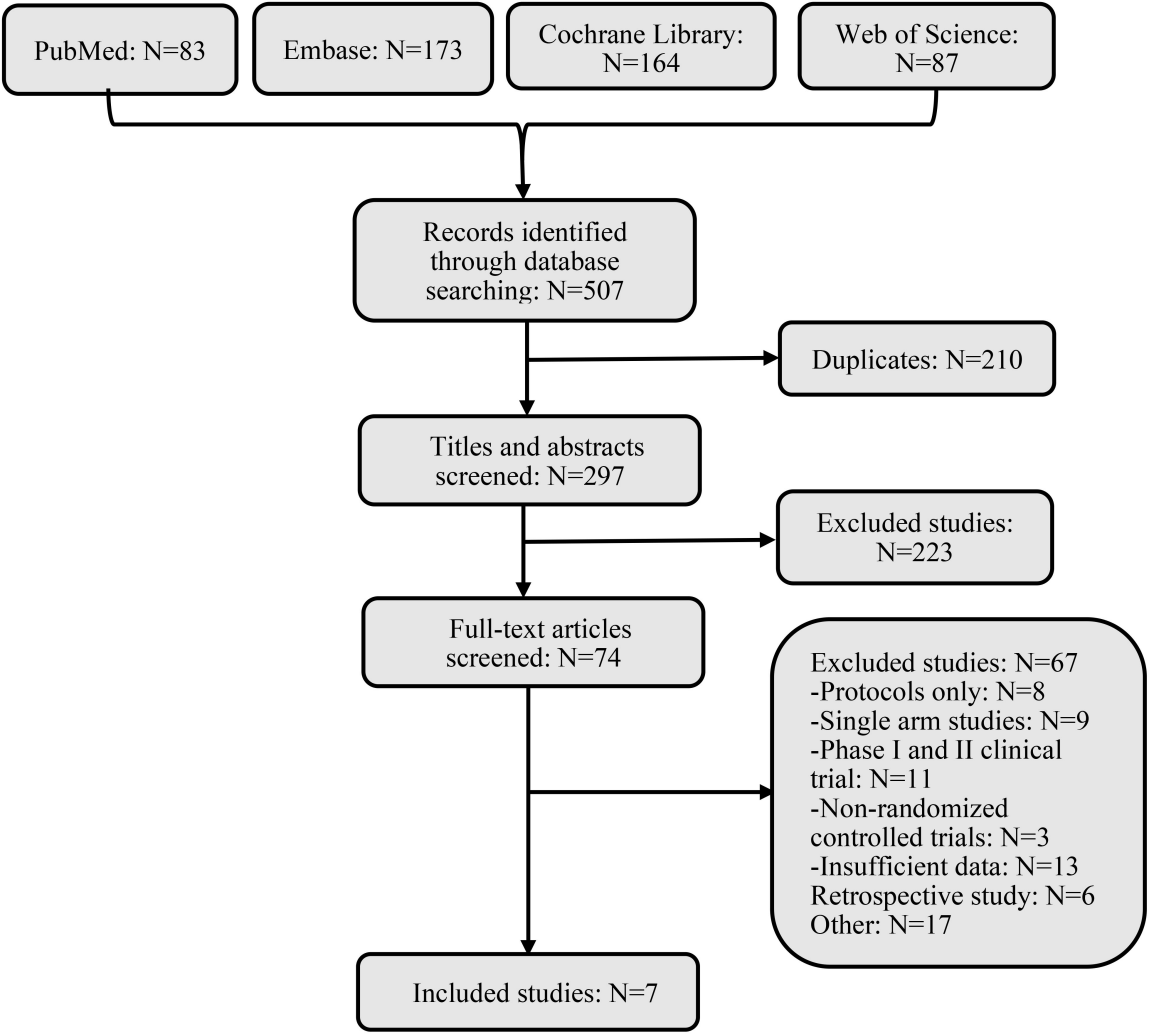
Flow diagram of literature and selections.

**Figure 2:**
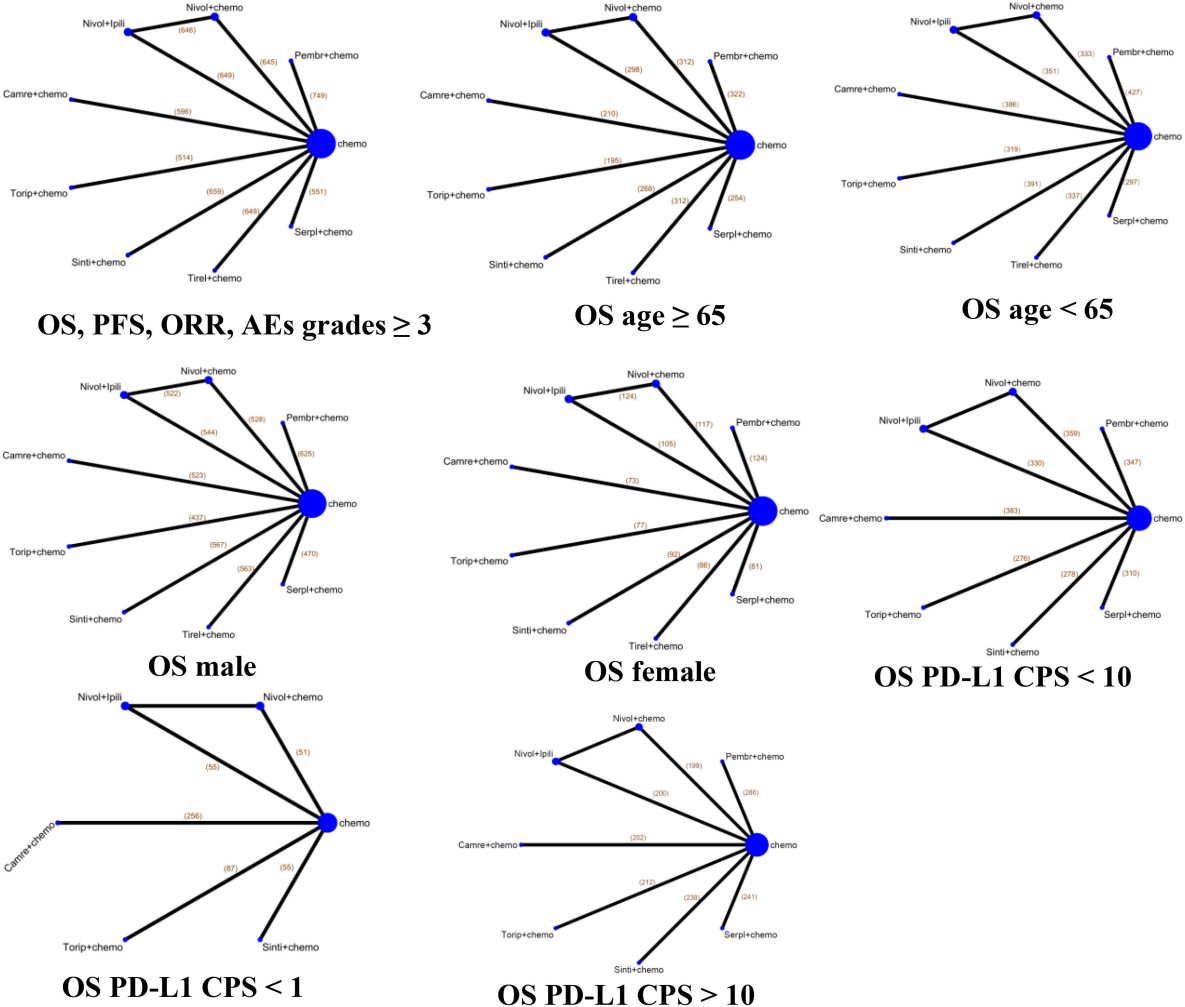
Network diagrams of comparisons of various treatments for overall survival, progression-free survival, objective response rate, and adverse events with grades ≥3 in advanced esophageal cancer, and different subgroups by age, gender, and PD-L1 status for OS in the enrolled study. The node indicates the kind of treatment, and the line shows the head-to-head comparison between two treatments. The thickness of line represents the number of trials in comparison with other treatments. Chemo, chemotherapy; Camre, camrelizumab; Nivol, nivolumab; Ipili, ipilimumab; Pembr, pembrolizumab; Serpl, serplulimab; Sinti, sintilimab; Tirel, tislelizumab; Torip, toripalimab. OS, overall survival; PFS, progression-free survival; ORR, objective response rate; AEs, adverse events.

We searched 507 records from four databases. By screening the titles and abstracts, 74 records were included, retrieved, and fully reviewed (**Figure 1**). After rigorous screening, we included seven phase III RCTs (14–19). A total of 4688 patients in the seven RCTs received nine diverse treatments including ICI + chemotherapy (pembrolizumab + chemotherapy, nivolumab + chemotherapy, tislelizumab + chemotherapy, toripalimab + chemotherapy, serplulimab + chemotherapy, camrelizumab + chemotherapy, sintilimab + chemotherapy), nivolumab + ipilimumab, and chemotherapy alone. **Figure 2** shows the networks, and the information on each enrolled study is listed in **Table 1**.

**Table 1:** Baseline characteristics of studies included in the network meta-analysis.

Study	Sample Size (No); median age	Female (%)	Intervention arm	Control arm	Key Eligibility Criteria	Reported outcomes
KEYNOTE-590	373/376 64/62	18/15	pembrolizumab 200mg d1 + chemotherapy (fluorouracil 800mg/m^2^ d1-5 + cisplatin 80mg/m^2^ d1[maximum of six cycles]) once every 3 weeks for up to 35 cycles	placebo d1 + chemotherapy (fluorouracil 800mg/m^2^ d1-5 + cisplatin 80mg/m^2^ d1[maximum of six cycles]) once every 3 weeks for up to 35 cycles	Locally advanced unresectable or metastatic EAC or ESCC or advanced/metastatic EGJ Siewert type 1 adenocarcinoma; treatment naïve; ECOG PS 0 or 1; measurable disease (RECIST v1.1)	Primary endpoint: OS and PFS by the investigator; Secondary endpoints: ORR by the investigator
ESCORT-1st	298/298 62/62	12.8/11.7	camrelizumab 200mg d1 + chemotherapy (paclitaxel 175mg/m^2^ d1 + cisplatin 75mg/m^2^ d1 [maximum of six cycles]) once every 3 weeks for up to disease progression	placebo d1 + chemotherapy (paclitaxel 175mg/m^2^ d1 + cisplatin 75mg/m^2^ d1 [maximum of six cycles]) once every 3 weeks for up to disease progression	Histologically or cytologically confirmed ESCC; advanced or metastatic ESCC; treatment naïve; ECOG PS 0 or 1; at least 1 measurable disease (RECIST v1.1)	Primary endpoint: PFS and OS by IRC; Secondary endpoints: PFS, ORR, DCR, DoR, OS rates, adverse events, and HRQoL by INV
CheckMate-648	321/325/324 64/63/64	21/17/15	nivolumab 240mg q2w (maximum of 2 years) + chemotherapy (fluorouracil 800mg/m^2^ d1-5 + cisplatin 80mg/m^2^ d1 q4w [maximum of six cycles]); nivolumab 3mg/kg q2w + ipilimumab 1mg/kg q6w (maximum of 2 years)	chemotherapy (fluorouracil 800mg/m^2^ d1-5 + cisplatin 80mg/m^2^ d1 q4w [maximum of six cycles])	Unresectable advanced, recurrent or metastatic ESCC; ECOG PS 0 or 1; no prior systemic treatment for advanced disease; measurable disease (RECIST v1.1)	Primary endpoint: OS and PFS (tumor cell PD-L1≥ 1%; Secondary endpoints: OS and PFS (all randomized); ORR (tumor cell PD-L1≥ 1% and all randomized
JUPITER-06	257/257 63/62	15.6/14.4	toripalimab 240mg d1 + chemotherapy (paclitaxel 175mg/m^2^ d1 + cisplatin 75mg/m^2^ d1 [maximum of six cycles]) once every 3 weeks for up to disease progression	placebo d1 + chemotherapy (paclitaxel 175mg/m^2^ d1 + cisplatin 75mg/m^2^ d1 [maximum of six cycles]) once every 3 weeks for up to disease progression	Histologically or cytologically confirmed advanced or metastatic ESCC; treatment-naïve for metastatic disease; ECOG PS 0 or 1; measurable disease per (RECIST v1.1)	Primary endpoint: PFS by BICR per RECIST v1.1 and OS; Secondary endpoints: PFS, ORR, DoR, DCR, and 1-year and 2-year PFS & OS rates, safety, and HRQoL by the investigator
ORIENT-15	327/332 63/63	15/13	sintilimab 200mg d1 q3w (maximum of 2 years) + chemotherapy (fluorouracil 800mg/m^2^ d1-5 or paclitaxel 87.5mg/m^2^ d1 d8 + cisplatin 75mg/m^2^ d1 q3w [maximum of six cycles])	placebo d1 q3w (maximum of 2 years) + chemotherapy (fluorouracil 800mg/m^2^ d1-5 or paclitaxel 87.5mg/m^2^ d1 d8 + cisplatin 75mg/m^2^ d1 q3w [maximum of six cycles])	Histologically or cytologically confirmed Locally advanced unresectable or recurrent or metastatic ESCC; ECOG PS 0 or 1; at least 1 measurable disease (RECIST v1.1)	Primary endpoint: OS (PD-L1 CPS≥ 10), OS (all randomized); Secondary endpoints: PFS, ORR, DCR, and DoR by the investigator
RATIONALE-306	326/323 64/65	13/13	tislelizumab 200mg d1 q3w (maximum of 2 years) + chemotherapy (maximum of six cycles) (cisplatin 60-80mg/m^2^ d1 or oxaliplatin 130mg/m^2^ d1) + (fluorouracil 750-800mg/m^2^ d1-5 or capecitabine 1000mg/m^2^ bid d1-14) or paclitaxel 175mg/m^2^ d1 q3w	placebo d1 q3w (maximum of 2 years) + chemotherapy (maximum of six cycles) (cisplatin 60-80mg/m^2^ d1 or oxaliplatin 130mg/m^2^ d1) + (fluorouracil 750-800mg/m^2^ d1-5 or capecitabine 1000mg/m^2^ bid d1-14) or paclitaxel 175mg/m^2^ d1 q3w	Locally advanced unresectable or recurrent or metastatic ESCC; no prior systemic treatment for advanced disease; ECOG PS 0 or 1; measurable disease per (RECIST v1.1)	Primary endpoint: OS (all randomized); Secondary endpoints: PFS, ORR, DoR, OS (PD-L1≥ 10%), HRQoL, and safety by the investigator
ASTRUM-007	368/183 64/64	14/16	serplulumab 3mg/kg d1 q3w (maximum of 2 years) + cisplatin 50mg/m2 d1 (maximum of 8 cycles) + fluorouracil 2400mg/m2 d1-2 (maximum of 12 cycles) q3w	placebo d1 q3w (maximum of 2 years) + cisplatin 50mg/m2 d1 (maximum of 8 cycles) + fluorouracil 2400mg/m2 d1-2 (maximum of 12 cycles) q3w	Histologically or cytologically confirmed Locally advanced unresectable or recurrent or metastatic ESCC; no prior systemic treatment for advanced disease; at least 1 measurable disease (RECIST v1.1); PD-L1 CPS≥ 1	Primary endpoint: PFS by IRRC per RECIST v1.1, and OS; Secondary endpoints: PFS by the investigator, ORR, DoR, and safety etc.

### 3.2 Network meta-analysis in the total population of ESCC with immunotherapy

**Figure 3:**
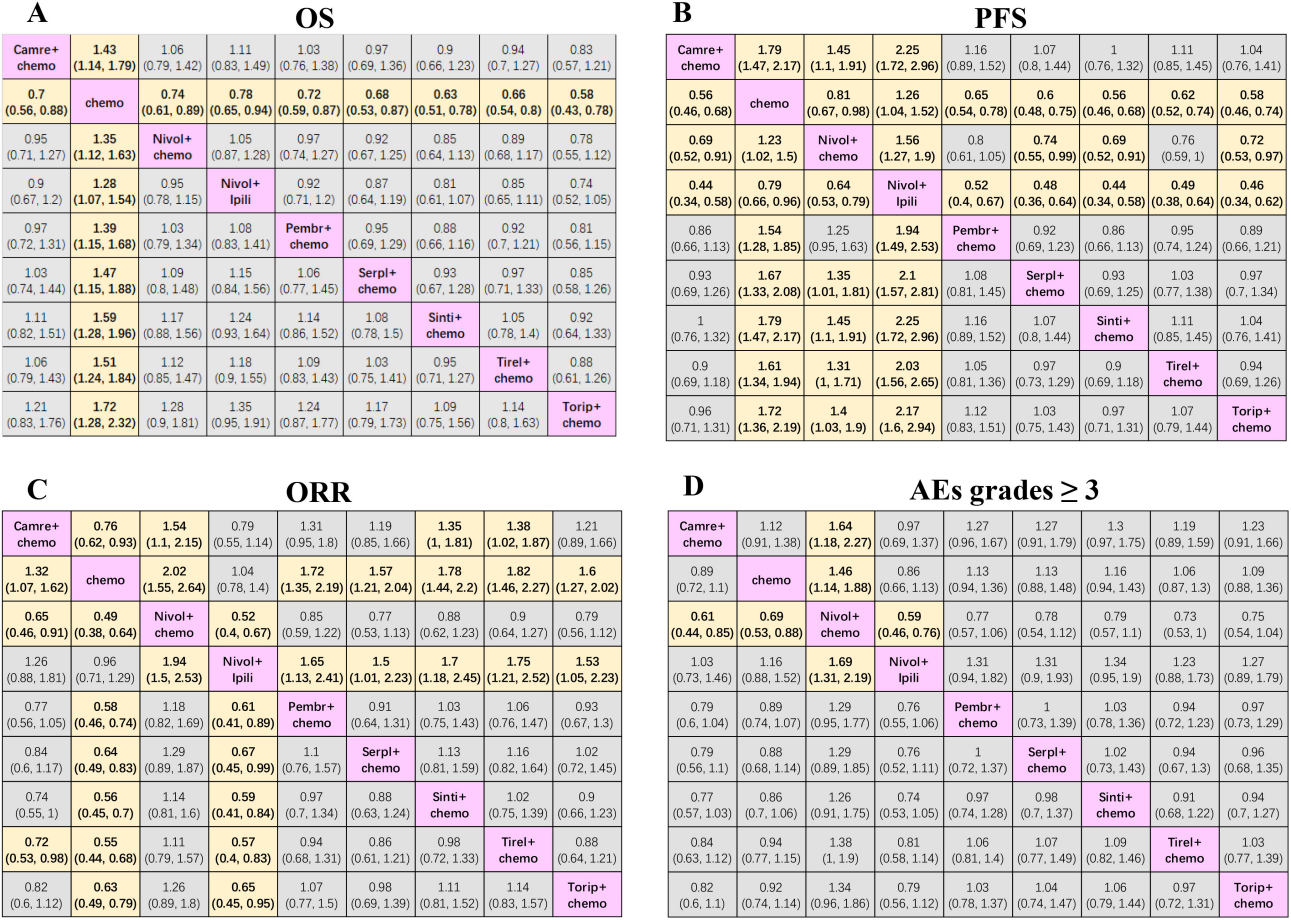
Efficacy and safety league table of the network meta-analysis. **(A)** Hazard ratios and 95% CIs for overall survival. **(B)** Hazard ratios and 95% CIs for progression-free survival. **(C)** Odds ratios and 95% CIs for objective response rate. **(D)** Odds ratios and 95% CIs for grades ≥ 3 adverse events of the network meta-analysis. Chemo, chemotherapy; Camre, camrelizumab; Nivol, nivolumab; Ipili, ipilimumab; Pembr, pembrolizumab; Serpl, serplulimab; Sinti, sintilimab; Tirel, tislelizumab; Torip, toripalimab. OS, overall survival; PFS, progression-free survival; ORR, objective response rate; AEs, adverse events; CI, confidence interval.

Our NMAs for OS, PFS, ORR, and grade ≥3 AEs in all patients included nine treatments (**Figure 2**). We present HRs or ORs of the total population in **Figure 3**. In terms of OS (**Figure 3A**), compared with the chemotherapy group, toripalimab + chemotherapy (0.58; 0.43, 0.78), sintilimab + chemotherapy (0.63; 0.51, 0.78), tislelizumab + chemotherapy (0.66; 0.54, 0.80), serplulimab + chemotherapy (0.68; 0.53, 0.87), camrelizumab + chemotherapy (0.70; 0.56, 0.88), pembrolizumab + chemotherapy (0.72; 0.59, 0.87), nivolumab + chemotherapy (0.74; 0.61, 0.89), and nivolumab + ipilimumab (0.78; 0.65, 0.94), all presented significantly better survival benefits.

In terms of PFS (**Figure 3B**) compared with nivolumab + chemotherapy, sintilimab + chemotherapy (0.69; 0.52, 0.91), camrelizumab + chemotherapy (0.69; 0.52, 0.91), toripalimab + chemotherapy (0.72; 0.53, 0.97), and serplulimab + chemotherapy (0.74; 0.55, 0.99) significantly prolonged the PFS. Compared with nivolumab + ipilimumab, sintilimab + chemotherapy (0.44; 0.34, 0.58), camrelizumab + chemotherapy (0.44; 0.34, 0.58), toripalimab + chemotherapy (0.46; 0.34, 0.62), serplulimab + chemotherapy (0.48; 0.36, 0.64), tislelizumab + chemotherapy (0.49; 0.38, 0.64), pembrolizumab + chemotherapy (0.52; 0.40, 0.67), and nivolumab + chemotherapy (0.64; 0.53, 0.79) showed the best survival benefits. In addition, sintilimab + chemotherapy (0.56; 0.46, 0.68), camrelizumab + chemotherapy (0.56; 0.46, 0.68), toripalimab + chemotherapy (0.58; 0.46, 0.74), serplulimab + chemotherapy (0.60; 0.48, 0.75), tislelizumab + chemotherapy (0.62; 0.52, 0.74), pembrolizumab + chemotherapy (0.65; 0.54, 0.78), and nivolumab + chemotherapy (0.81; 0.67, 0.98) showed better efficacy than chemotherapy alone. Conversely, although nivolumab + ipilimumab could prolong OS compared to chemotherapy, it failed to prolong PFS and even had poorer efficacy than chemotherapy (1.26; 1.04, 1.52).

In terms of ORR (**Figure 3C**), nivolumab + chemotherapy (0.65; 0.46, 0.91) and tislelizumab + chemotherapy (0.72; 0.53, 0.98) improved ORR relative to camrelizumab + chemotherapy. Compared with nivolumab + ipilimumab, nivolumab + chemotherapy (0.52; 0.40, 0.67), tislelizumab + chemotherapy (0.57; 0.40, 0.83), sintilimab + chemotherapy (0.59; 0.41, 0.84), pembrolizumab + chemotherapy (0.61; 0.41, 0.89), toripalimab + chemotherapy (0.65; 0.45, 0.95), and serplulimab + chemotherapy (0.67; 0.45, 0.99) also significantly improved ORR. Additionally, nivolumab + chemotherapy (0.49; 0.38, 0.64), tislelizumab + chemotherapy (0.55; 0.44, 0.68), sintilimab + chemotherapy (0.56; 0.45, 0.70), pembrolizumab + chemotherapy (0.58; 0.46, 0.74), toripalimab + chemotherapy (0.63; 0.49, 0.79), serplulimab + chemotherapy (0.64; 0.49, 0.83), and camrelizumab + chemotherapy (0.76; 0.62, 0.93) were superior to chemotherapy alone.

In terms of AEs with grades ≥3 (**Figure 3D**), the results showed that nivolumab + chemotherapy resulted in a higher probability of AEs of grade ≥3 in comparison with nivolumab + ipilimumab (1.69; 1.31, 2.19), camrelizumab + chemotherapy (1.64; 1.18, 2.27), and chemotherapy alone (1.46; 1.14, 1.88).

### 3.3 Subgroup-level network meta-analysis for OS by age, gender, and PD-L1 expression

**Figure 4:**
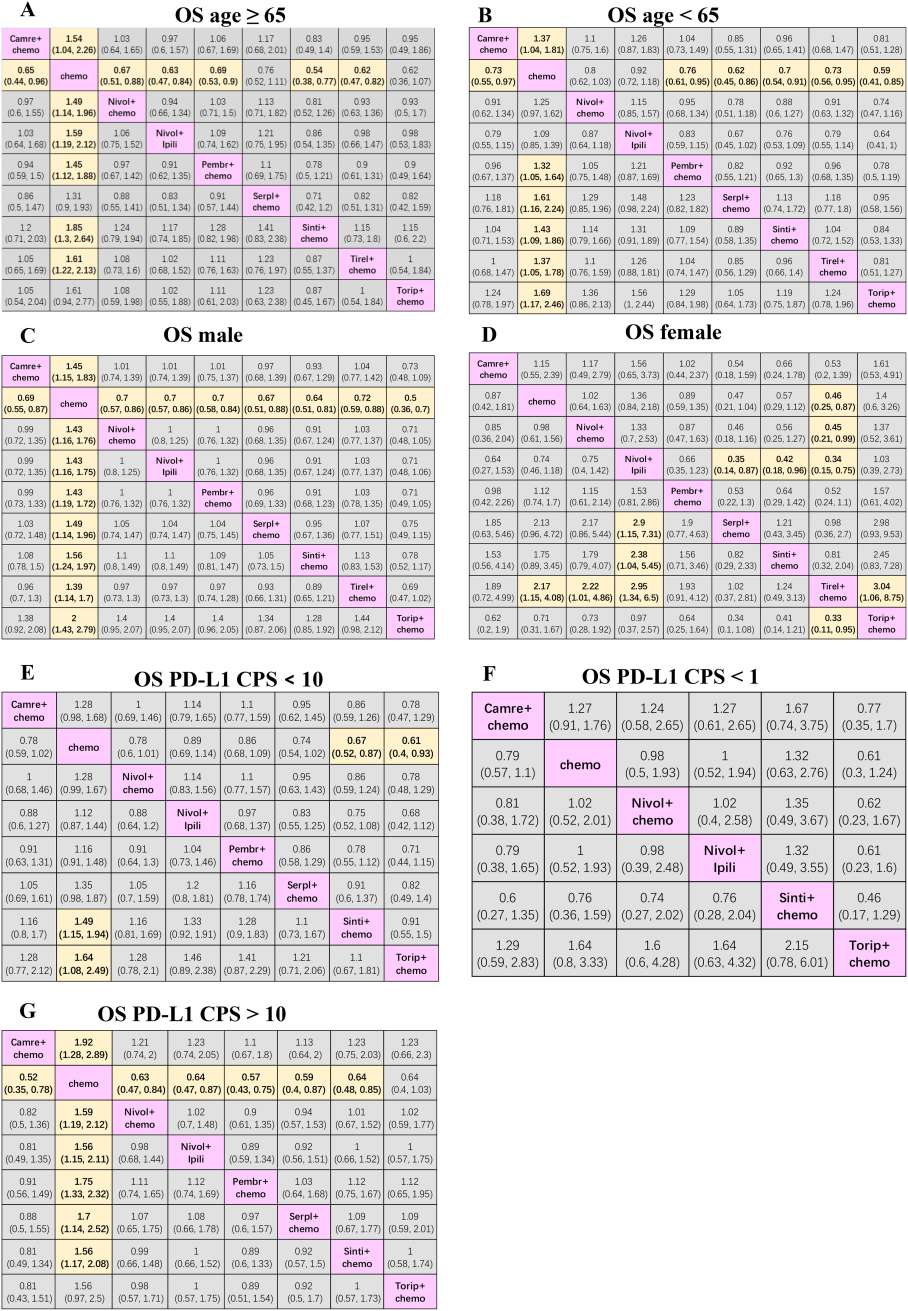
Efficacy and safety league table of the network meta-analysis. **(A)** HRs and 95% CIs for overall survival in patients aged **≥** 65. **(B)** HRs and 95% CIs for OS in patients aged **<** 65. **(C)** HRs and 95% CIs for OS in male patients. **(D)** HRs and 95% CIs for OS in female patients. **(E)** HRs and 95% CIs for OS in patients with PD-L1 CPS < 10. **(F)** HRs and 95% CIs for OS in patients with PD-L1 CPS < 1. **(G)** HRs and 95% CIs in patients with PD-L1 CPS > 10. Chemo, chemotherapy; Camre, camrelizumab; Nivol, nivolumab; Ipili, ipilimumab; Pembr, pembrolizumab; Serpl, serplulimab; Sinti, sintilimab; Tirel, tislelizumab; Torip, toripalimab. OS, overall survival; HRs, Hazard ratios; CI, confidence interval.

Regarding all subgroups, the NMAs for OS included nine treatments. In patients ≥65 years of age (**Figure 4A**), sintilimab + chemotherapy (0.54; 0.38, 0.77), tislelizumab + chemotherapy (0.62; 0.47, 0.82), nivolumab + ipilimumab (0.63; 0.47, 0.84), camrelizumab + chemotherapy (0.65; 0.44, 0.96), nivolumab + chemotherapy (0.67; 0.51, 0.88), and pembrolizumab + chemotherapy (0.69; 0.53, 0.90) resulted in higher OS compared with chemotherapy alone.

In patients <65 years of age (**Figure 4B**), toripalimab + chemotherapy (0.59; 0.41, 0.85), serplulimab + chemotherapy (0.62; 0.45, 0.86), sintilimab + chemotherapy (0.70; 0.54, 0.91), tislelizumab + chemotherapy (0.73; 0.56, 0.95), camrelizumab + chemotherapy (0.73; 0.55, 0.97), and pembrolizumab + chemotherapy (0.76; 0.61, 0.95) were more effective than chemotherapy alone.

In males (**Figure 4C**), compared with the chemotherapy group, toripalimab + chemotherapy (0.50; 0.36, 0.70), sintilimab + chemotherapy (0.64; 0.51, 0.81), serplulimab + chemotherapy (0.67; 0.51, 0.88), camrelizumab + chemotherapy (0.69; 0.55, 0.87), nivolumab + ipilimumab (0.70; 0.57, 0.86), nivolumab + chemotherapy (0.70; 0.57, 0.86), pembrolizumab + chemotherapy (0.70; 0.58, 0.84), and tislelizumab + chemotherapy (0.72; 0.59, 0.88), all showed significantly better efficacies. In females (**Figure 4D**), tislelizumab + chemotherapy provided a better survival benefit compared with toripalimab + chemotherapy (0.33; 0.11, 0.95), nivolumab + ipilimumab (0.34; 0.15, 0.75), nivolumab + chemotherapy (0.45; 0.21, 0.99), and chemotherapy alone (0.46; 0.25, 0.87). Additionally, serplulimab + chemotherapy (0.35; 0.14, 0.87) and sintilimab + chemotherapy (0.42; 0.18, 0.96) presented better survival compared with nivolumab + ipilimumab.

Where PD-L1 CPS <10 (**Figure 4E**), sintilimab + chemotherapy (0.67; 0.52, 0.87) and toripalimab + chemotherapy (0.61; 0.40, 0.93) ensured better survival than chemotherapy alone. For patients with PD-L1 CPS <1 (**Figure 4F**), adding immunotherapy to chemotherapy did not improve OS compared to chemotherapy alone. For patients with PD-L1 CPS > 10 (**Figure 4G**), compared with chemotherapy alone, both immunotherapy combined with chemotherapy and double immunotherapy can significantly improve the OS of patients with advanced esophageal cancer, except for toripalimab + chemotherapy.

### 3.4 Ranking probabilities

**Figure 5:**
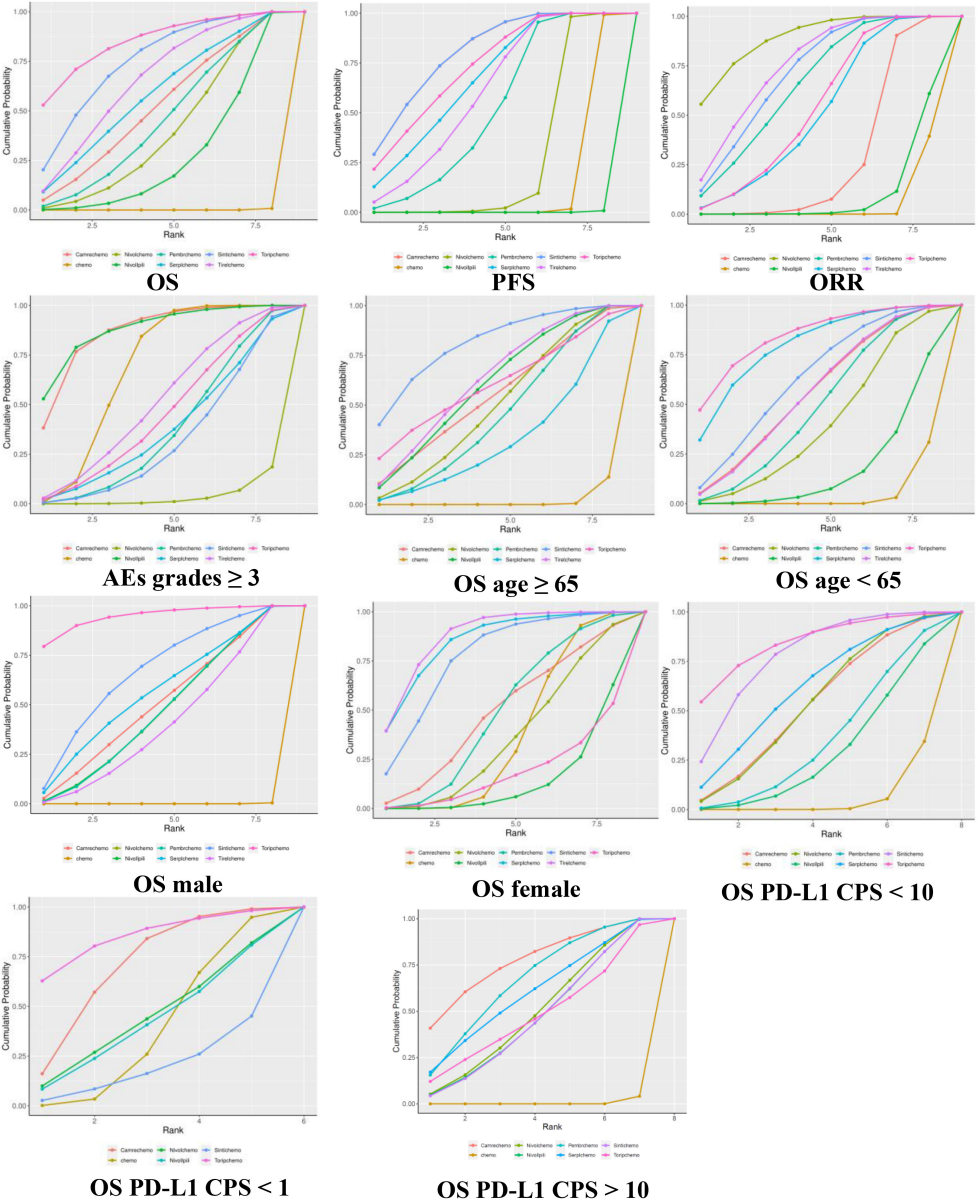
Cumulative ranking probabilities of treatment. The surface under the cumulative ranking curve is the probability that a particular treatment is among the best of those in the network, with larger values representing higher ranking probabilities. Chemo, chemotherapy; Camre, camrelizumab; Nivol, nivolumab; Ipili, ipilimumab; Pembr, pembrolizumab; Serpl, serplulimab; Sinti, sintilimab; Tirel, tislelizumab; Torip, toripalimab.

The Bayesian ranking profiles for the survival and safety of the immunotherapies in the total population and various subgroups are shown in **Figure 5**.

Toripalimab + chemotherapy and sintilimab + chemotherapy had the highest (SUCRA 85%) and second highest (75%) probabilities, respectively, in ranking best for improving OS. Sintilimab + chemotherapy and camrelizumab + chemotherapy showed the highest (80%) probability of ranking first in extending PFS. Nivolumab + chemotherapy presented the highest (89%) probability of ranking first for improving ORR. In addition, nivolumab + ipilimumab (88%) had the least toxicity and the best safety profile, whereas camrelizumab + chemotherapy (86%) had similar probabilities of ranking last in causing AEs of grade ≥3.

Toripalimab + chemotherapy showed the highest probability of ranking first for prolonging OS in PD-L1 CPS < 1 (85%), PD-L1 CPS <10 (84%), males (95%), and patients aged <65 (84%). Tislelizumab + chemotherapy presented the highest probability of ranking best for improving OS in subgroups of females (87%). Additionally, sintilimab + chemotherapy showed the highest probability of ranking first for prolonging OS in patients >65 years of age.

### 3.5 Subgroup analysis of OS in females, and patients with PD-L1 CPS < 1 or 10

**Figure 6:**
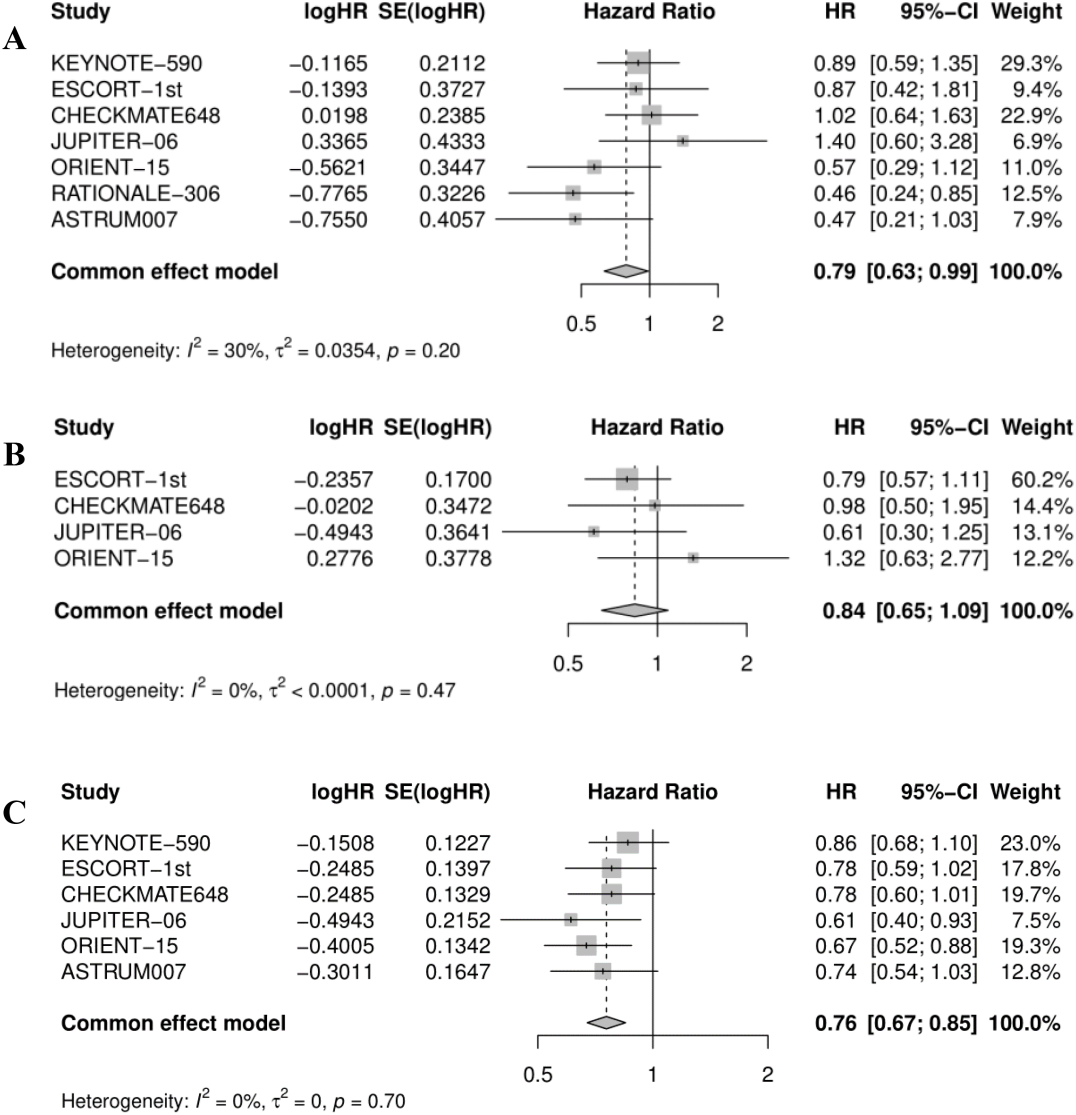
Forest plot for **(A)** overall survival in female, **(B)** OS in patients with PD-L1 CPS <1, and **(C)** OS in patients with PD-L1 CPS <10. OS, overall survival.

Approximately 70% of esophageal cancer diagnoses occur in men, and there is a two-fold to three-fold difference in incidence and mortality rates between the sexes (20). Rates of esophageal cancer are higher in developing versus developed countries for men but are comparable for women (21). The data from several RCTs indicated that the majority of female patients with esophageal cancer did not benefit from immunotherapy combined with chemotherapy except for tislelizumab + chemotherapy (0.46; 0.24, 0.85). Due to the limited number of female patients included in these studies, further quantitative analysis is needed to verify efficacy. Compared with the chemotherapy group, immunotherapy combined with chemotherapy reduced the risk of death in female esophageal cancer patients by 21% (pooled HR: 0.79, 95% CI: 0.63-0.99, I^2^ = 30%; **Figure 6A**). The heterogeneity of the combined results of the seven studies was small.

The benefits from immunotherapy are enhanced in esophageal cancer tumors with elevated levels of PD-L1 expression with respect to CPS (7, 22). Four RCTs indicated that none of the immunotherapy plus chemotherapy regimens improved OS for ESCC patients with PD-L1 CPS <1. Combining the effect values of the data from four studies, showed the same results (pooled HR: 0.84, 95% CI: 0.65-1.09, I^2^ = 0%; **Figure 6B**), which meant that adding immunotherapy to chemotherapy did not improve the efficacy. As displayed in **Figure 6C**, immunotherapy plus chemotherapy improved OS relative to chemotherapy alone (pooled HR: 0.76, 95% CI: 0.67-0.85, I^2^ = 0%).

## 4 Discussion

Patients with esophageal cancer that is metastatic or unresectable and cannot be treated with curative-intent chemoradiotherapy (CRT) have a poor prognosis; survival in clinical trials has historically been <1 year (23). However, the use of immunotherapy with chemotherapy has recently improved survival for this patient group (24). The recent advent of immunotherapy has opened up new possibilities for ESCC patients. Due to the multitude of treatment strategies and the lack of credible and direct head-to-head comparisons across all the effective therapeutic regimens, clinicians face serious challenges in formulating treatment decisions. In this study, we performed a Bayesian network meta-analysis to analyze the efficacy and safety in advanced or metastatic ESCC patients who received immune checkpoint inhibitors along with chemotherapy as first-line treatments.

In general, all the combination immunotherapy-chemotherapy regimens were significantly superior to standard chemotherapy. This study may help oncologists determine the optimal choice of ICIs in advanced ESCC. Toripalimab + chemotherapy had the highest probability of presenting the best OS for the total population relative to chemotherapy, followed by sintilimab + chemotherapy and tislelizumab + chemotherapy. Toripalimab + chemotherapy also showed the highest probability of ranking first in prolonging OS in most of the subgroups (males, patients aged <65 years, and patients with PD-L1 CPS <1 or 10). Sintilimab + chemotherapy showed the highest probability of ranking first in improving OS in females. Additionally, tislelizumab + chemotherapy presented the highest probability of ranking first for improving efficacy in patients ≥65 years of age.

In terms of PFS, sintilimab + chemotherapy showed the best benefit over chemotherapy and nivolumab + ipilimumab presented the lowest survival benefit in patients with advanced ESCC. In addition, camrelizumab + chemotherapy had the highest probability of ranking first in prolonging PFS in the subgroup of patients with PD-L1 CPS >1; serplulimab + chemotherapy was most likely to be ranked first for PFS in the subgroup of patients with PD-L1 CPS >10 (Supplementary). Nivolumab + chemotherapy showed the highest probability of ranking first in improving ORR in the overall population.

ESCC represents a significant disease burden in the older population. It is a highly morbid condition with the older population being especially vulnerable to the complications of dysphagia, malnutrition and sarcopenia (25). Advancing age is accompanied by decreases in the number, function, and activity of immune cells, cytokines and other immunoregulatory molecules (26). This review shows that ICIs have similar efficacy and safety in elderly cancer patients compared to younger patients. All combination therapy regimens have better efficacy than chemotherapy alone for esophageal cancer patients over 65 years of age. Nivolumab + ipilimumab was most likely to be ranked third for OS in patients aged ≥65. The survival benefits may be related to the low toxicity of dual drug immunotherapy. However, for esophageal cancer patients under the age of 65, Nivolumab + ipilimumab was only superior to chemotherapy alone in terms of efficacy ranking, and there was no statistically significant difference compared to chemotherapy. In addition, a lower radiological response rate was noted for nivolumab + ipilimumab compared with chemotherapy alone or nivolumab + chemotherapy, and there is a risk of early progression and death for patients treated without chemotherapy, resulting in a lower grade of recommendation compared with nivolumab + chemotherapy (20). But for ESCC patients with chemotherapy contraindications, older age, and refusal to undergo chemotherapy, nivolumab + ipilimumab can be recommended.

Research on immunotherapy has made significant progress in esophageal cancer and is gradually rewriting the global treatment model for it. However, only a portion of esophageal cancer patients can significantly benefit from immunotherapy, so screening potential beneficiaries of ICIs is currently an important challenge. The expression level of PD- L1 protein in esophageal cancer is closely related to the efficacy of ICIs and is currently the most important prognostic marker for efficacy. The KEYNOTE-590, ESCORT-1st, CHECKMATE-648, and ASTRUM-007 trials showed that ICIs combined with chemotherapy did not benefit patients with ESCC with low PD-L1 expression (CPS<10). However, the JUPITER-06 and ORIENT-15 trials indicated that sintilimab or toripalimab plus chemotherapy could improve survival benefits of ESCC patients with low PD-L1 expression (CPS<10) compared to chemotherapy. Therefore, a much-debated question is whether patients with ESCC with low PD-L1 expression will truly benefit from PD-1 antibody plus chemotherapy (27). Based on the results of this study, the pooled analysis of patients with low PD-L1 expression (CPS<10) revealed significant clinical benefit from the addition of PD-1 antibody to chemotherapy, but pooled analysis adding PD-1 antibody to chemotherapy did not improve the OS of patients with PD-L1-CPS<1.

In terms of safety, nivolumab + ipilimumab and camrelizumab + chemotherapy may be safer choices than other combined treatment strategies, with a relatively lower incidence of grade ≥3 AEs. Nivolumab + chemotherapy was the only option that could increase the incidence of adverse reactions compared to chemotherapy. Although treatment-related AEs were more common with the nivolumab-based regimens than with chemotherapy alone, treatment-related AEs of grade 3 or 4 that had potential immunologic causes occurred in no more than 6% of the patients across the organ categories. There can be significant differences in the response of patients to immunotherapy. Some patients may be more tolerant of treatment, while others may experience serious adverse reactions. This depends on many factors, including the individual’s immune system status, disease type, and medication. Patients should know potential side effects, how to monitor their symptoms, and when to seek medical help.

## 5 Limitations

Currently, clinicians treating ESCC encounter challenges in devising treatment plans, as there is no reliable and direct head-to-head comparison of all known effective treatment options for advanced diseases. To facilitate a comparative analysis of practical information across different treatment modalities, we conducted a network meta-analysis and examined various systemic treatment options for first-line use in the treatment of advanced ESCC. However, our network meta-analysis has several limitations. First, this study is not intended to replace primary prospective evidence, but to provide additional data to assist clinical decision-making. The heterogeneity of the studies, including the different stratification factors, molecular tumor characteristics, chemotherapy regimens, and the different geographical sources of registered populations, could jeopardize the general clinical applicability of individual research findings. Second, patients were not stratified based on factors such as race, which may alter treatment outcomes, and the efficacy of immunotherapy combined with chemotherapy in Asian populations may differ from that in Western populations. Third, to facilitate a fair comparison of the effectiveness of various treatment options, we assume all chemotherapy regimens to be equally effective. This assumption, however, heightens the risk of bias, thus the results must be interpreted with utmost caution. Therefore, further direct comparative studies are needed to more accurately evaluate the efficacy of different ICI regimens.

## 6 Conclusions

In conclusion, the combination of ICIs with chemotherapy provided a high probability of more effective treatment in comparison with chemotherapy alone for patients with ESCC. In this network meta-analysis, toripalimab and sintilimab plus chemotherapy were ranked as providing the highest OS and PFS benefit in the first-line setting, respectively. Our NMA provides a data-rich, up-to-date perspective on the role of first-line treatment options in advanced ESCC, making it a useful, evidence-based source of guidance to assist in treatment decision-making for advanced ESCC.

## Data Availability

The original contributions presented in the study are included in the article/supplementary material. Further inquiries can be directed to the corresponding authors.
